# SARS-CoV-2 Breakthrough Infections among US Embassy Staff Members, Uganda, May–June 2021

**DOI:** 10.3201/eid2806.220427

**Published:** 2022-06

**Authors:** Julie R. Harris, Daniel Owusu, Kevin O’Laughlin, Adam L. Cohen, Amen Ben Hamida, Jaymin C. Patel, Molly M. Freeman, Thomas Nsibambi, Rebecca Nieves, Barbara J. Marston, Samuel Wasike, Jennifer S. Galbraith, Amy L. Boore, Lisa J. Nelson, Sarah Anne J. Guagliardo, John D. Klena, Ketan Patel, Marek Ma

**Affiliations:** Centers for Disease Control and Prevention, Kampala, Uganda (J.R. Harris, T. Nsibambi, S. Wasike, J.S. Galbraith, A.L. Boore, L.J. Nelson);; Centers for Disease Control and Prevention, Atlanta, Georgia, USA (D. Owusu, K. O’Laughlin, A.L. Cohen, A.B. Hamida, J.C. Patel, M.M. Freeman, B.J. Marston, S.A.J. Guagliardo, J.D. Klena, K. Patel);; US Department of State, Kampala (R. Nieves, M. Ma)

**Keywords:** COVID-19, respiratory infections, severe acute respiratory syndrome coronavirus 2, SARS-CoV-2, SARS, coronavirus disease, zoonoses, viruses, coronavirus, vaccine, breakthrough, US Embassy, Uganda

## Abstract

The SARS-CoV-2 Delta variant emerged shortly after COVID-19 vaccines became available in 2021. We describe SARS-CoV-2 breakthrough infections in a highly vaccinated, well-monitored US Embassy community in Kampala, Uganda. Defining breakthrough infection rates in highly vaccinated populations can help determine public health messaging, guidance, and policy globally.

Breakthrough infections after vaccination are a known phenomenon, but the expected rate of such infections for SARS-CoV-2 was unknown in mid-2021. This uncertainty was compounded by the emergence of the SARS-CoV-2 Delta variant, initially recognized in India in October 2020 (*1*) and identified in the United States in March 2021 (*2*). 

During February–June 2021, the US Embassy in Kampala, Uganda, offered COVID-19 vaccinations to the US mission community, including US citizens working at the US Embassy (14%), their eligible family members (11%), locally employed staff (i.e., Ugandan citizens working at the US Embassy) (64%), and other eligible persons. Of the 833 persons eligible for vaccination through the US Embassy, 94% were fully vaccinated by April 18, 2021, and 97% were fully vaccinated by May 23, 2021. By May 2021, Uganda was entering its second major wave of SARS-CoV-2 infections, dominated by the Delta variant (*3*). Throughout the pandemic, mission community members were required to report SARS-CoV-2 infections and symptoms suggestive of infection, and free SARS-CoV-2 testing was available at the US Embassy. 

During May 24–June 26, 2021, a total of 20 PCR-confirmed SARS-CoV-2 infections were reported to the health unit at the US Embassy in Kampala among the mission community and in 1 US government staff member temporarily stationed in Uganda for work, an attack rate of 2.3% (Figure). Among 19 infected persons who consented to use of their data, 8 (42%) were female; mean age was 40 (range 18–63) years. Ten (53%) persons identified as Black African, 6 (32%) as White American, 2 (11%) as Asian American, and 1 (5%) as Black American. Thirteen (68%) persons were symptomatic, and 4 (21%) cases were identified through travel-related testing. Nine (47%) of the infected persons had contact with a person with confirmed SARS-CoV-2 infection before their symptom onset; 3 of the contacts were also fully vaccinated, 2 with Moderna (https://www.modernatx.com) and 1 with AstraZeneca (https://www.astrazeneca.com) vaccines. Four persons had underlying conditions, including diabetes, hypertension, chronic hepatitis B virus infection, and pregnancy. No infected persons were seriously ill or required hospitalization.

**Figure:**
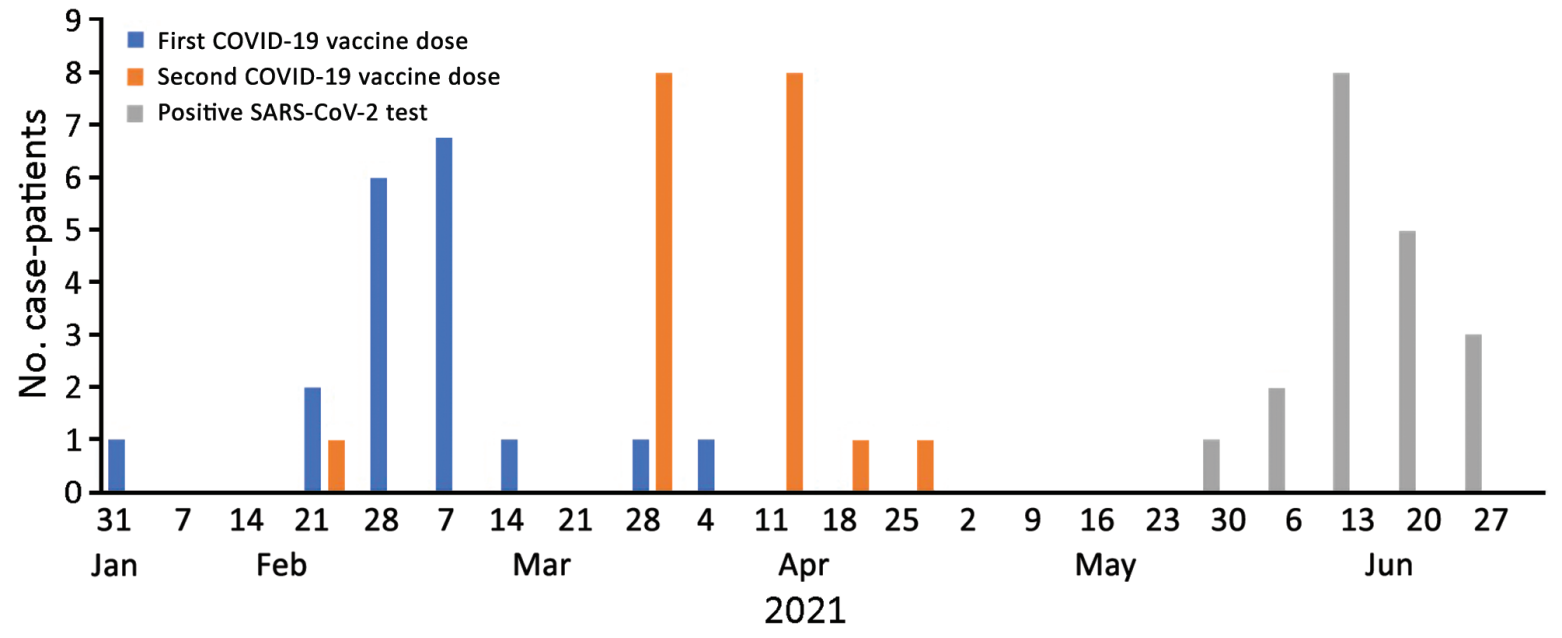
Dates of first COVID-19 vaccine dose, second vaccine dose, and positive SARS-CoV-2 test among 19 staff, family members, and visiting US government staff with identified postvaccination SARS-CoV-2 infection, US Embassy, Kampala, Uganda, 2021. Among 20 persons with breakthrough infections, 19 consented to have their data included.

All infected persons were fully vaccinated at the time of symptom onset or positive SARS-CoV-2 test results. Seventeen infected persons had received 2 doses of the Moderna vaccine at the US Embassy, and all 17 received the second dose during March 27–April 23, 2021 (Figure). Two persons had received vaccines outside the US Embassy: 1 person received 2 Moderna doses in the United States, the second of which they received on February 22; the other person received an initial Pfizer-BioNTech (https://www.pfizer.com) vaccine dose in Uganda and a second dose in the United States on May 1. The mean time between first and second dose for all 19 patients was 28.9 (range 21–35) days. The mean time between second dose and positive SARS-CoV-2 test was 66 (range 47–107) days.

Although breakthrough infections are expected with any vaccine, the rate of SARS-CoV-2 breakthrough infections, and the potential for transmission from fully vaccinated persons, was not clear at the time we began our investigation. An article describing breakthrough infections in the United States during January–April 2021, immediately before the Delta wave in the United States, stated that only a small fraction of vaccinated persons experience breakthrough infections and that these persons account for a small percentage of all COVID-19 cases (*4*). Those authors reported ≈10,000 breakthrough cases, which were passively reported from states, in a population of 101 million vaccinated persons, a breakthrough rate of 0.01%. Although the authors acknowledged those findings were certainly an undercount, identifying true rates of breakthrough infections in terms of vaccination status and reporting rates in an extremely large and diverse population is difficult, if not impossible. Our findings showed that breakthrough infections could be expected early after SARS-CoV-2 vaccines became available, even in highly vaccinated populations, when community transmission is high and provided early suggestive evidence of transmission from fully vaccinated, infected persons. Because new variants of concern can rapidly emerge, evaluation of closely monitored populations with known, consistently recorded vaccination histories and rapid access to diagnostic testing, such as embassy communities, could be useful for determining health messaging and preventive measures in areas that do not yet have exposure to the variants. Instances of well-described sentinel populations are rare but can be highly informative (*5*). 

We shared our experience in real time with other embassies in the region to help examine common experiences and guide expectations. As future SARS-CoV-2 variants emerge, identifying novel, rapid approaches to monitor breakthrough infection rates continually and systematically is critical to determining vaccine effectiveness and the need for additional interventions to reduce disease spread. Well-delineated populations with relatively homogeneous vaccination practices and clear documentation of vaccination histories, such as overseas US Embassy staff or similar populations, could be a useful source of data for public health prevention and control efforts during pandemics.
